# Infection Masquerading As Spontaneous Tumor Lysis Syndrome

**DOI:** 10.7759/cureus.40503

**Published:** 2023-06-16

**Authors:** Jen DeSalvo, Allison L Rossetti

**Affiliations:** 1 Internal Medicine - Pediatrics, The Ohio State University, Nationwide Children's Hospital, Columbus, USA

**Keywords:** tumor lysis syndrome, oncologic emergency, systemic infection, bacteremia, endocarditis, spontaneous tumor lysis syndrome

## Abstract

Tumor lysis syndrome (TLS) is a life-threatening condition due to malignant tumor cell lysis resulting in severe metabolic derangements that require prompt recognition and management to prevent progression to end-organ damage and death. This case describes a patient presenting with clinical and laboratory abnormalities complicated by multi-organ dysfunction concerning spontaneous TLS from a suspected undiagnosed malignancy. The patient had an unremarkable malignancy workup and was ultimately diagnosed with endocarditis, which improved with treatment of the infection. Therefore, this case demonstrates that systemic infections may rarely present with metabolic abnormalities and multi-organ dysfunction resembling spontaneous TLS.

## Introduction

Tumor lysis syndrome (TLS) is a life-threatening condition affecting patients with rapidly proliferating malignancies [1]. Characterized by malignant tumor cell lysis and release of intracellular contents into the bloodstream, TLS results in severe metabolic derangements, primarily hyperuricemia, hyperkalemia, hyperphosphatemia, and hypocalcemia. If not promptly recognized and treated with hypouricemic drugs and hyperhydration, this may rapidly progress to end-organ damage, including acute kidney injury (AKI), arrhythmia, and seizure, as well as death [1-3]. In patients with spontaneous TLS from an undiagnosed malignancy, early recognition of TLS may facilitate prompt identification of malignancy and early initiation of therapy to prevent subsequent morbidity and mortality [2]. It is imperative, however, that internists maintain a broad differential diagnosis and avoid anchoring bias in patients presenting with laboratory abnormalities seemingly classic for TLS. Systemic infections may, in rare cases, present with metabolic derangements and multi-organ dysfunction resembling spontaneous TLS.

## Case presentation

A 57-year-old male with a history of heart failure with preserved ejection fraction, mitral regurgitation, and atrial fibrillation on rivaroxaban presented to the emergency department with two weeks of dyspnea on exertion and one month of night sweats, fevers, and chills. He had a tooth extraction two weeks prior to presentation and completed treatment with antibiotics. Physical exam was remarkable for tachypnea and known holosystolic murmur (temperature 97.1 ⁰ Fahrenheit, heart rate 93 beats per minute, respiratory rate 28 breaths per minute, blood pressure 109/75 mm Hg, SpO2 97% on room air). Initial labs showed lactic acidosis, hyperkalemia, hyperphosphatemia, hypocalcemia, acute kidney injury (AKI), leukocytosis, acute normocytic anemia, elevated brain natriuretic peptide, and troponinemia (Table 1).

**Table 1:** Patient's initial investigations

Lab	Value	Reference range
Potassium	5.7 mmol/L	3.5 - 5.0 mmol/L
Phosphorus	7.2 mg/dL	2.2 - 4.6 mg/dL
Calcium	8.5 mg/dL	8.6 - 10.5 mg/dL
Creatinine	2 mg/dL	0.70 - 1.30 mg/dL
White blood cell count	28 K/uL	3.73 - 10.10 K/uL
Hemoglobin	10.7 g/dL (14.4 g/dL eight months prior)	13.4 - 16.8 g/dL
Platelet count	284 K/uL	146 - 337 K/uL
Brain natriuretic peptide	944 pg/mL	0 - 100 pg/mL
Troponin	263 ng/L	<53 ng/L
Lactate	17 mmol/L	0.5 - 1.6 mmol/L

Electrocardiogram demonstrated prolonged QT interval corrected for heart rate (511 ms) without ischemic changes. Chest X-ray was unremarkable. Further workup in the setting of these metabolic abnormalities and B symptoms on hospital admission showed hyperuricemia, elevated lactate dehydrogenase, indirect hyperbilirubinemia, acute liver injury (ALI), coagulopathy, and unremarkable fibrinogen and haptoglobin (Table 2).

**Table 2:** Patient's additional investigations given suspicion of spontaneous tumor lysis syndrome

Lab	Value	Reference range
Uric acid	13.5 mg/dL	3.5 - 7.0 mg/dL
Lactate dehydrogenase	4493 U/L	100 - 190 U/L
Total bilirubin	2.4 mg/dL	<1.5 mg/dL
Direct bilirubin	0.8 mg/dL	<0.3 mg/dL
Alanine transaminase	3510 U/L	10 - 52 U/L
Aspartate aminotransferase	4047 U/L	10 - 39 U/L
International normalized ratio	14.5	0.9 - 1.1
Fibrinogen	407 mg/dL	220 - 410 mg/dL
Haptoglobin	172 mg/dL	44 - 215 mg/dL

He was treated with hyperhydration, allopurinol, and rasburicase given concern for spontaneous TLS from suspected undiagnosed malignancy. Malignancy evaluation including peripheral blood smear, differential, alpha fetoprotein assay, serum protein electrophoresis, immunophenotyping, and computed tomography scans of the head, chest, abdomen, and pelvis were unremarkable, and patient deferred endoscopic assessment. Infectious workup including testing for respiratory viruses and atypical bacteria, cytomegalovirus, Epstein-Barr virus, varicella zoster virus, herpes simplex virus, hepatitis, immunoglobulins, autoimmune antibodies, panorex, and renal and abdominal ultrasounds were unremarkable.

**Figure 1:**
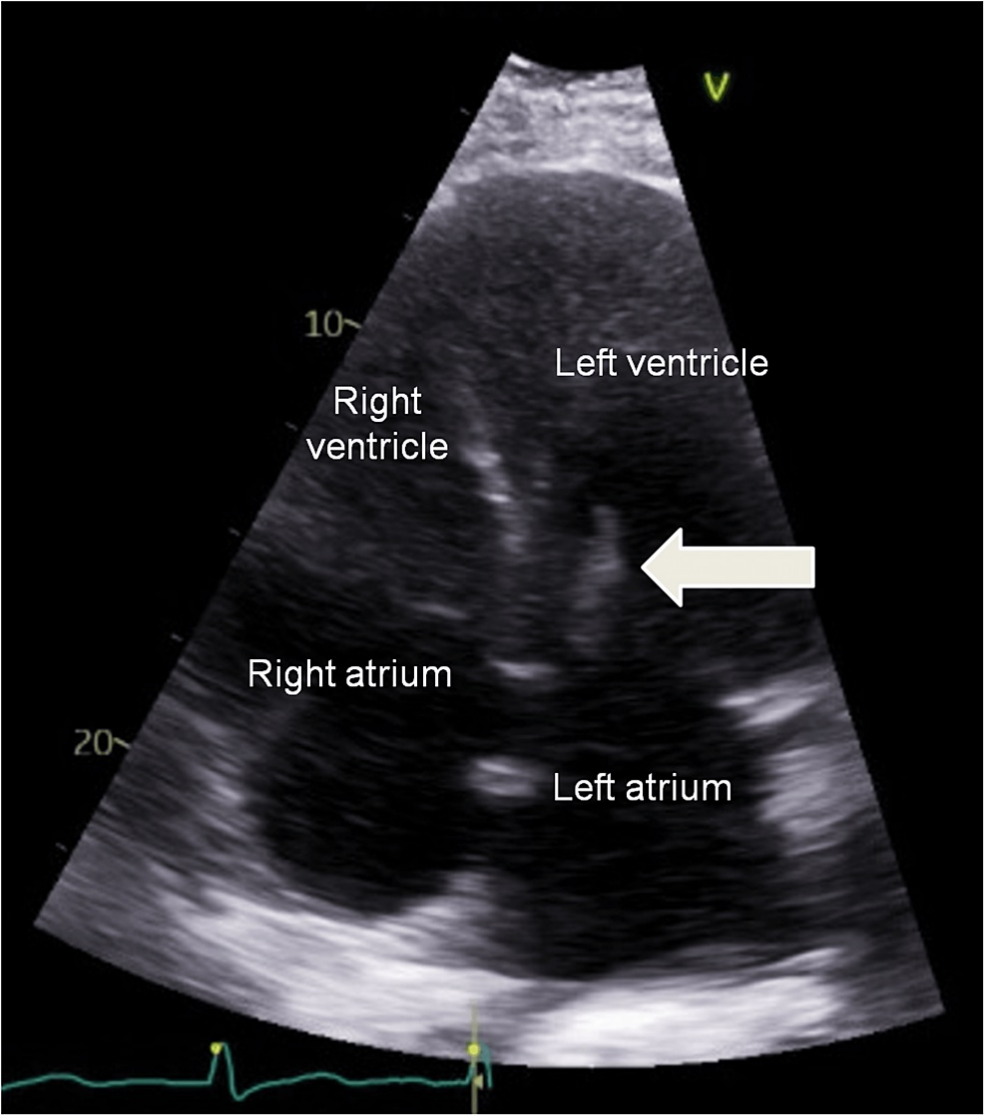
Transthoracic echocardiogram showing mitral valve vegetation, as indicated by the arrow, concerning for endocarditis

On the second day of hospital admission, blood cultures grew Staphylococcus caprae. Echocardiogram demonstrated mitral valve endocarditis not amenable to surgical intervention (Figure 1). The patient was discharged with six weeks of targeted antibiotic therapy.

Given his clinical and laboratory improvement with treatment of infection and supportive therapies within four days of hospital admission (Table 3), his laboratory derangements were attributed to a robust inflammatory response with high white blood cell turnover in the setting of systemic infection with sequelae of AKI and ALI causing metabolic abnormalities and coagulopathy, complicated by supratherapeutic levels of rivaroxaban.

**Table 3:** Patient's additional investigations on the fourth day of hospital admission

Lab	Value	Reference range
Potassium	3.6 mmol/L	3.5 - 5.0 mmol/L
Phosphorus	2.5 mg/dL	2.2 - 4.6 mg/dL
Calcium	7.8 mg/dL	8.6 - 10.5 mg/dL
Creatinine	1.7 mg/dL	0.70 - 1.30 mg/dL
White blood cell count	15.3 K/uL	3.73 - 10.10 K/uL
Hemoglobin	9.7 g/dL	13.4 - 16.8 g/dL
Platelet count	160 K/uL	146 - 337 K/uL
Lactate	1.7 mmol/L	0.5 - 1.6 mmol/L
Uric acid	2.4 mg/dL	3.5 - 7.0 mg/dL
Lactate dehydrogenase	534 U/L	100 - 190 U/L
Total bilirubin	2.2 mg/dL	<1.5 mg/dL
Direct bilirubin	1 mg/dL	<0.3 mg/dL
Alanine transaminase	2216 U/L	10 - 52 U/L
Aspartate aminotransferase	858 U/L	10 - 39 U/L
International normalized ratio	4.3	0.9 - 1.1
Fibrinogen	227 mg/dL	220 - 410 mg/dL

Of note, on outpatient clinic follow-up three months after hospital admission, subsequent screening for prostate cancer was unremarkable (prostate-specific antigen 0.2 ng/mL, reference range 0.0-3.9 ng/mL), and screening for colorectal cancer via colonoscopy had been scheduled, although not yet completed.

## Discussion

This case is a novel presentation of infection with initial clinical and laboratory abnormalities resembling spontaneous TLS, which has been previously unreported in the scientific literature to our knowledge. Given its associated morbidity and mortality, rapid recognition of TLS and prompt initiation of empiric treatment are paramount [1-3]. TLS may occur in hematologic or solid malignancies after initiation of cytotoxic therapy [3]. However, spontaneous TLS may occur prior to initiation of chemotherapy in undiagnosed pre-existing malignancies with high rates of cell proliferation or large tumor burden [2]. In this case, the patient initially presented with B symptoms in addition to hyperuricemia, hyperkalemia, hyperphosphatemia, hypocalcemia, AKI, and ALI, resulting in timely treatment with hypouricemic agents and hyperhydration given concern for spontaneous TLS. Fortunately, this patient's malignancy workup was unremarkable during admission. However, he deferred inpatient upper and lower endoscopies and was not screened for prostate cancer. Prior case reports have demonstrated evidence of spontaneous TLS in patients with previously undiagnosed metastatic colorectal and prostate cancer [4-6].

Furthermore, this case is significant given it illustrates the importance of avoiding anchoring bias and premature closure while maintaining a broad differential diagnosis when initiating therapies in patients presenting with laboratory abnormalities seemingly classic for TLS. This is crucial given similar metabolic derangements and end-organ dysfunction may occur in robust inflammatory responses secondary to infectious, inflammatory, or toxin-mediated insults, as exemplified in this case. Prompt diagnosis and treatment in this atypical presentation of infection are especially important given that prior studies in patients with sepsis have demonstrated that hyperuricemia, hyperphosphatemia, hypophosphatemia, and hypocalcemia have been associated with increased mortality [7-9]. Further research is needed to determine if drug intervention to normalize these laboratory abnormalities in patients will reduce morbidity and mortality.

## Conclusions

Spontaneous TLS results in metabolic abnormalities in patients with undiagnosed hematologic and solid malignancies, which may lead to morbidity and mortality if not promptly recognized and treated. This case describes a patient presenting with clinical and laboratory abnormalities complicated by multi-organ dysfunction resembling spontaneous TLS. The patient had an unremarkable malignancy workup and was ultimately diagnosed with endocarditis. Given they improved with the treatment of infection, their initial presentation was attributed to a robust inflammatory response to systemic infection complicated by multi-organ dysfunction resulting in metabolic abnormalities.
